# Multicentre derivation and validation of a colitis-associated colorectal cancer risk prediction web tool

**DOI:** 10.1136/gutjnl-2020-323546

**Published:** 2021-05-14

**Authors:** Kit Curtius, Misha Kabir, Ibrahim Al Bakir, Chang Ho Ryan Choi, Juanda L Hartono, Michael Johnson, James E East, Oxford IBD Cohort Study Investigators, James O Lindsay, Roser Vega, Siwan Thomas-Gibson, Janindra Warusavitarne, Ana Wilson, Trevor A Graham, Ailsa Hart

**Affiliations:** 1 Centre for Genomics and Computational Biology, Barts Cancer Institute, Queen Mary University of London, London, UK; 2 Division of Biomedical Informatics, Department of Medicine, University of California San Diego, La Jolla, California, USA; 3 Department of Surgery and Cancer, Imperial College London, London, UK; 4 Department of Gastroenterology, St Mark's Hospital and Academic Institute, London, UK; 5 Department of Gastroenterology & Hepatology, St George Hospital, Sydney, New South Wales, Australia; 6 Division of Gastroenterology, National University Hospital, Singapore; 7 Department of Medicine, Yong Loo Lin School of Medicine, National University of Singapore, Singapore; 8 Translational Gastroenterology Unit, Nuffield Department of Medicine, John Radcliffe Hospital, Oxford, UK; 9 Oxford NIHR Biomedical Research Centre, University of Oxford, Oxford, UK; 10 Blizard Institute, Barts and The London School of Medicine and Dentistry, London, UK; 11 Department of Gastroenterology, Royal London Hospital, Barts Health NHS Trust, London, UK; 12 Department of Gastroenterology, University College London Hospitals NHS Foundation Trust, London, UK; 13 Department of Metabolism, Digestion and Reproduction, Imperial College London, London, UK; 14 Colorectal Surgery and Lennard-Jones Intestinal Failure Unit, St Mark’s Hospital and Academic Institute, London, UK

**Keywords:** ulcerative colitis, colorectal cancer, clinical decision making, dysplasia

## Abstract

**Objective:**

Patients with ulcerative colitis (UC) diagnosed with low-grade dysplasia (LGD) have increased risk of developing advanced neoplasia (AN: high-grade dysplasia or colorectal cancer). We aimed to develop and validate a predictor of AN risk in patients with UC with LGD and create a visual web tool to effectively communicate the risk.

**Design:**

In our retrospective multicentre validated cohort study, adult patients with UC with an index diagnosis of LGD, identified from four UK centres between 2001 and 2019, were followed until progression to AN. In the discovery cohort (n=246), a multivariate risk prediction model was derived from clinicopathological features using Cox regression. Validation used data from three external centres (n=198). The validated model was embedded in a web tool to calculate patient-specific risk.

**Results:**

Four clinicopathological variables were significantly associated with AN progression in the discovery cohort: endoscopically visible LGD >1 cm (HR 2.7; 95% CI 1.2 to 5.9), unresectable or incomplete endoscopic resection (HR 3.4; 95% CI 1.6 to 7.4), moderate/severe histological inflammation within 5 years of LGD diagnosis (HR 3.1; 95% CI 1.5 to 6.7) and multifocality (HR 2.9; 95% CI 1.3 to 6.2). In the validation cohort, this four-variable model accurately predicted future AN cases with overall calibration Observed/Expected=1.01 (95% CI 0.64 to 1.52), and achieved 100% specificity for the lowest risk group over 13 years of available follow-up.

**Conclusion:**

Multicohort validation confirms that patients with large, unresected, multifocal LGD and recent moderate/severe inflammation are at highest risk of developing AN. Personalised risk prediction provided via the Ulcerative Colitis-Cancer Risk Estimator (*www.UC-CaRE.uk*) can support treatment decision-making.

**Significance of this study**

**What is already known on this subject?**

- The risk of UC-associated low-grade dysplasia (LGD) progression to more advanced neoplasia is currently not clearly defined. The literature consists of historical data from small heterogeneous observational studies with limited follow-up or lack of information on endoscopic resection status.

**What are the new findings?**

- We present the results from the largest multicentre cohort study to evaluate LGD long-term prognosis based on clinicopathological factors that are reflective of modern surveillance techniques.
- Recent moderate or severe active inflammation or LGD that is large, not fully resectable or is multifocal remain independent predictors of advanced neoplasia progression, even when stratified to reflect the most modern era of high-definition endoscopic imaging, chromoendoscopy and advanced polypectomy techniques.
- Colorectal cancer incidence after endoscopic resection of unifocal polypoid and non-polypoid dysplasia is 0.6 per 100 patient-years.
- Long-term incidence of advanced neoplasia is similar if LGD is invisible or if LGD is visible but not completely endoscopically resected.
- Using these data, we have designed and externally validated a cancer risk prediction tool for patients with UC with LGD.

**Significance of this study**

**How might it impact on clinical practice in the foreseeable future?**

- The Ulcerative Colitis-Cancer Risk Estimator tool can be used to calculate and communicate individualised numerical cancer risk estimates to patients with colitis with LGD.
- It facilitates the risk stratification of the lowest risk patients, who can be reassured by undergoing continued surveillance, and those at the highest risk who may benefit from a prophylactic colectomy.
- This visual aid presents the calculated risk in a graphical and pictorial form to optimise risk comprehension and informed treatment choices.

## Introduction

Patients with ulcerative colitis (UC) have an increased lifetime risk of developing colorectal cancer (CRC) and of CRC-related death.^1–3^ Consequently, patients with UC are advised to engage in a colonoscopic surveillance programme 8–10 years after diagnosis to detect and resect any dysplasia before it progresses to adenocarcinoma.^4–7^ Due to a high CRC risk, high-grade dysplasia (HGD) warrants preventive colectomy surgery or *en bloc* endoscopic resection with intensive surveillance follow-up if unifocal.^4–7^ The natural history of low-grade dysplasia (LGD) progression is less well defined with a wide range of rates reported for progression of LGD lesions to advanced neoplasia (AN; HGD or CRC) due to the inclusion of historical data from small population studies with heterogeneous terminology and limited follow-up.^8^ The quality of endoscopic surveillance has also evolved over the past three decades with standardisation of surveillance technique, advances in imaging technology such as high-definition white-light imaging and chromoendoscopy, and resection techniques such as endoscopic mucosal resection and submucosal dissection. These advances have been linked with higher rates of visible dysplasia detection, lower proportions of dysplasia categorised as ‘invisible’ and lower AN progression rates.^8–10^ A recent systematic review of studies from the videoendoscopic era has found wide variation in AN progression rates after endoscopic resection from 0% to 23% at 5 years for polypoid LGD and 0% to 22% at 2 years for non-polypoid LGD.^8^ A large observational study evaluating the effect of endoscopic resection on long-term LGD prognosis is required to be able to better inform patients of their cancer risk if they continue surveillance rather than have a colectomy.

Patients can be reluctant to consider surgical management even when the risks of CRC are high due to concerns about the negative impact that complications, stoma or ileoanal pouch function may have on their quality of life, given that they are often in clinical remission at the time of dysplasia detection.^11–13^ Shared clinician–patient decision-making is particularly important when the evidence and best management option is unclear and there are potentially harmful consequences associated with the choice that is eventually made. This is the case for management of LGD in UC: the risks and consequences of developing CRC despite surveillance must be balanced against having a life-changing surgical operation. Providing evidence-based and individualised numerical CRC risk estimates using visual decision aids can promote patient engagement with decision-making.^13–15^

We aimed to evaluate the impact of endoscopic resection of LGD on risk of future AN in the largest multicentre cohort study of this to date, and to identify the factors that can predict AN progression in the 21st century. Our objective was to develop and validate an online simple and visual multivariate risk prediction model to communicate patient-specific risk, following the Transparent Reporting of a Multivariable Prediction Model for Individual Prognosis or Diagnosis guideline.^16^ With our model, we created the Ulcerative Colitis-Cancer Risk Estimator (*UC-CaRE*) web-based application that is publicly accessible at www.uc-care.uk and can be used by clinicians to aid neoplasia risk communication, patient education and shared decision-making.

## Materials and methods

### Study design and patient cohort identification

**Figure 1:**
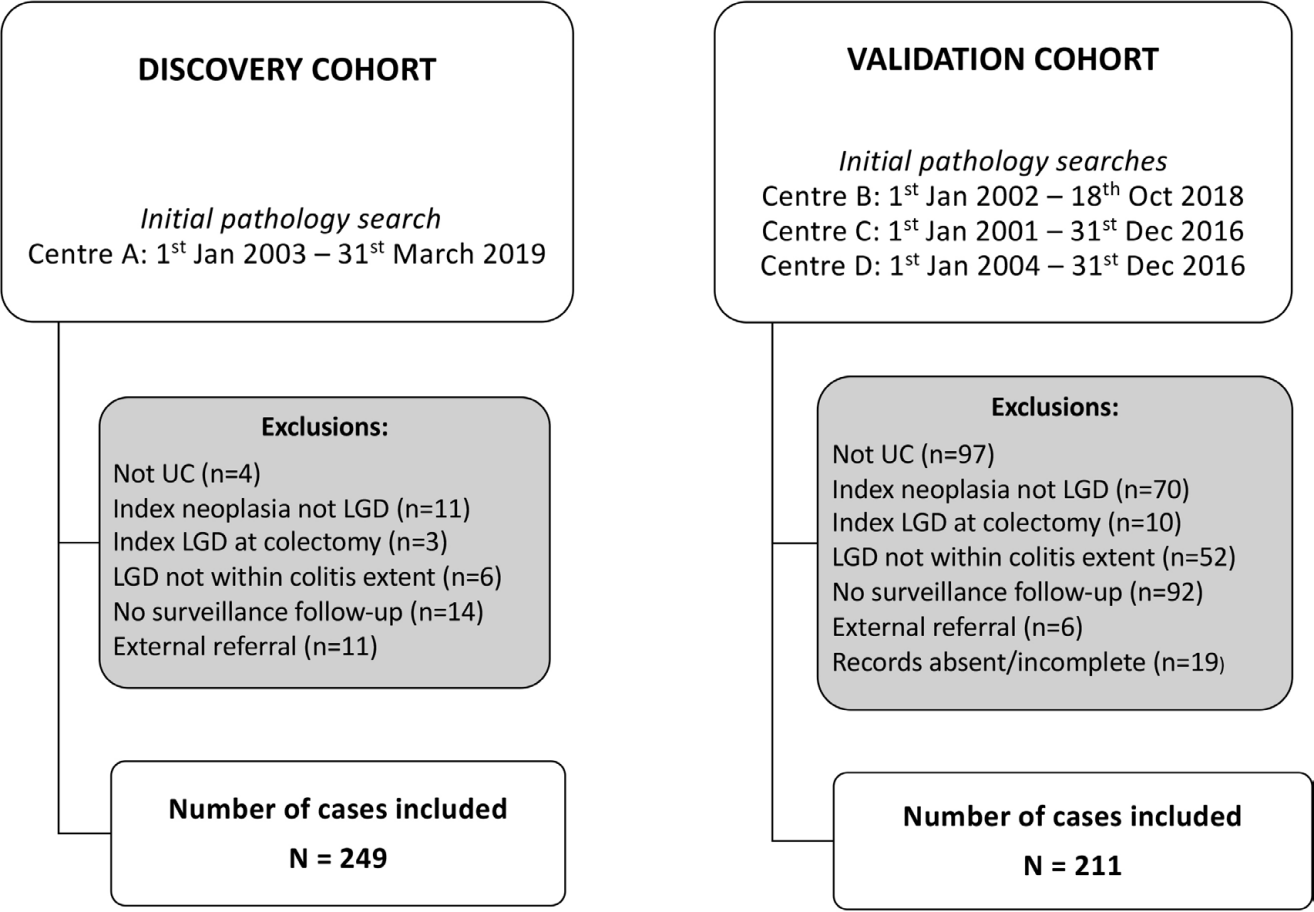
Flow chart of low-grade dysplasia (LGD) cases included and excluded (centre details in online supplemental table S1).

A retrospective cohort study of patients with UC diagnosed with an index case of LGD at four UK hospitals was undertaken. Hospital pathology databases were searched using the following terms to identify patients with UC who had been diagnosed with LGD: ‘ulcerative colitis’ or ‘inflammatory bowel disease’ and ‘dysplasia’, ‘low-grade dysplasia’, ‘adenocarcinoma’ or ‘dysplasia associated mass lesion’. The searched time periods were marginally different between each site and started between 1 January 2001 and 1 January 2004 and ended between 31 December 2016 and 31 March 2019 (figure 1).

10.1136/gutjnl-2020-323546.supp1 Supplementary data

**Figure 2:**
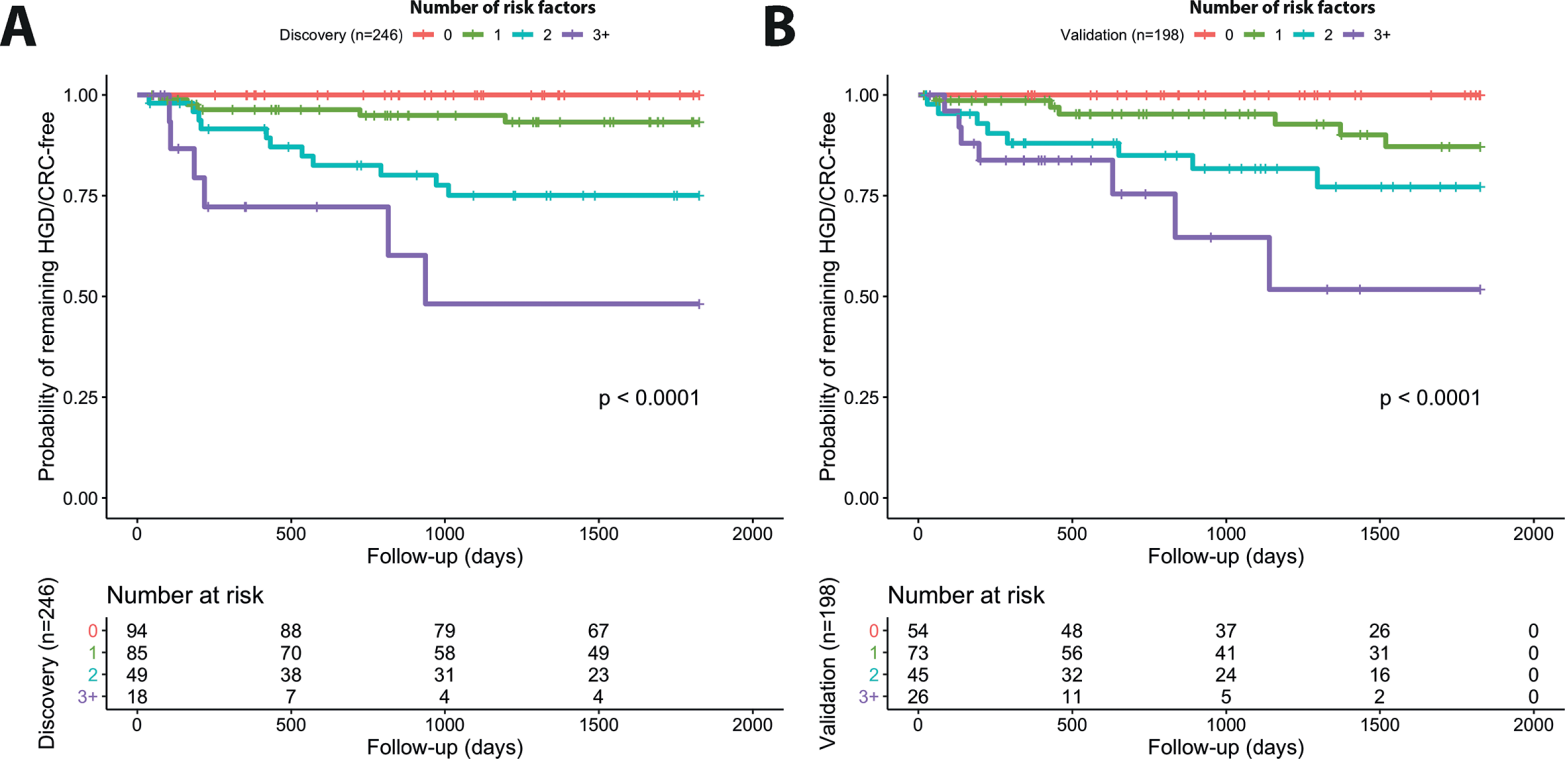
Kaplan-Meier plots for probability of remaining free of high-grade dysplasia (HGD) or colorectal cancer (CRC) to assess risk stratification and predictive power of multivariate model. (A) Discovery (n=246) and (B) validation (n=198) cohorts stratified by risk score (0 to 3+) defined by final multivariate model at index low-grade dysplasia (LGD) diagnosis to year 5 follow-up (see online supplemental figure S3 for similar results over total years of follow-up).

#### Inclusion criteria

We included adult patients (aged 18 years or over) with histologically confirmed UC who had an index LGD diagnosis and had at least one follow-up examination of the whole colon after the index LGD diagnosis, either by colonoscopy or pathological analysis of a surgical colectomy specimen. The index LGD diagnosis was interpreted as the first documentation of LGD to have: (1) been confirmed by a second GI histopathologist; (2) developed within the known histological extent of colitis based on historical pathology reports; and (3) resection status known by judgement of the histopathologist/endoscopist.

#### Exclusion criteria

Patients were excluded if they had a diagnosis of Crohn’s disease, IBD unclassified or indeterminate colitis, there was no adequate follow-up examination of the colon after the index LGD diagnosis, and the index LGD was: (1) located proximal to the known extent of historical microscopic inflammation as this was classed as a sporadic adenoma; (2) diagnosed after a proctocolectomy, that is, was first noted incidentally within the surgical colonic specimen; (3) diagnosed at the same time as or after another more advanced neoplastic lesion (either HGD or adenocarcinoma); (4) diagnosed at an external institution to one of the study centres and the exact date of onset was unclear.

### Data collection

The clinical notes, endoscopy and histology reporting systems at each centre were interrogated to collect data for clinicopathological variables including: concomitant primary sclerosing cholangitis (PSC, which had been radiologically or histologically confirmed); patient exposure to 5-aminosalicylate, immunomodulators (thiopurines or methotrexate) and biological medications; macroscopic morphology of the index LGD as per the Paris classification^17^ (polypoid, non-polypoid or invisible); size and location of the largest visible index LGD; multifocality; completion of any endoscopic resection undertaken; presence of any macroscopic or histological active inflammation in the colon at the time of or within the 5 years preceding the index LGD; any chronic features of inflammation (colonic stricture, postinflammatory polyps (PIP), scarred colon or a tubular and shortened colon); a previous diagnosis of indefinite for dysplasia; and use of chromoendoscopy during any surveillance colonoscopy performed before, at time of or after the index LGD diagnosis. Histological inflammation was categorised as moderate to severe active inflammation as defined by grade 3 or 4 of the Nancy histological index.^18^ This requires the presence of ulceration and/or moderate to severe acute inflammatory cells infiltrate (multiple clusters of neutrophils in the lamina propria or epithelium). As reporting of the Nancy histological index or other similar validated histological inflammation scores was not a standardised procedure across the centres in the study time period, a qualitative description correlating to Nancy histological index grade 3 or 4 was categorised as moderate to severe active inflammation. Cumulative inflammation burden (CIB), which has been shown to be an independent predictor of neoplasia incidence in IBD, was calculated as previously described^19^: namely as the sum of the average histological inflammation scores between each pair of surveillance episodes multiplied by the surveillance interval in years. A minimum of one documented colonic histological examination within 5 years prior to the index LGD diagnosis was required in order to be able to calculate a 5-year CIB.

Surveillance colonoscopy at each centre was performed in accordance with the national guidelines at the time,^7 20^ including the use of chromoendoscopy. Dysplasia was categorised as invisible if it was detected on random mucosal biopsy with absence of a corresponding visible lesion in a colonic segment with good bowel preparation. If the lesion was found to be visible on targeted colonoscopy re-examination within 3 months, the lesion categorisation was changed from invisible to visible polypoid or non-polypoid. When multiple LGD lesions were found, categorisation of the morphology was based on the lesion considered to have more carcinogenic potential (from an earlier St Mark’s cohort^21^) in descending order of non-polypoid, invisible and polypoid morphology. The index LGD was categorised as multifocal LGD if more than one LGD discrete visible lesion was detected on index colonoscopy, regardless of the colonic segment, or foci of invisible LGD were detected in more than one colonic segment. Endoscopic resection where possible was based on histological confirmation of complete endoscopic resection, but often this could not be confirmed due to piecemeal resection or diathermy artefact, so completion of resection was based on endoscopic criteria. Invisible LGD was categorised as not being endoscopically resected. If there was multifocal LGD, whereby a visible lesion was successfully endoscopically resected but there was another focus of invisible LGD, this LGD case was categorised as not being successfully endoscopically resected. A lesion was considered a PIP if only inflammatory and/or granulation tissue and no neoplastic tissue was detected histologically within the lesion. Patients were recorded as having multiple PIPs if the endoscopist reported on there being ‘a few’, ‘several’, ‘many’ or ‘multiple’ PIPs within the colon. Colonic scarring was based on the endoscopist’s documentation of its macroscopic appearance.

### Follow-up outcomes

End of surveillance follow-up was determined by the date of the first incidence of AN (either HGD or CRC) or censoring at the last surveillance colonoscopy or proctocolectomy date.

### Statistical analysis of patient cohorts

The St Mark’s cohort of patients was used as the discovery set, and the patient cohorts from the three other centres were pooled together to form a validation set. Differences between the clinical characteristics of the cohorts were assessed using χ^2^ tests for categorical variables and Mann-Whitney U tests for non-parametric continuous variables (significance required p<0.002 with Bonferroni multiple testing correction). Data analysis was performed using SPSS (IBM SPSS Statistics for Macintosh, V.25.0). Incidence rates of AN with 95% CI were determined using OpenEpi software.^22^

### Statistical model selection and validation

In the discovery set, 17 clinicopathological variables were tested for association with time to progression to AN using univariate Cox proportional hazards (PH) models (significance required p<0.003 with Bonferroni multiple testing correction). Significantly associated variables were included in a multivariate Cox PH model, and individual patient risk scores were computed. Kaplan-Meier (KM) estimation and log-rank tests were used to compare survival between dichotomised risk groups in discovery and validation sets. Positive and negative predictive values (PPV/NPV respectively) were assessed from KM curves to evaluate the predictive power in the validation set. Survival analysis was carried out using the *survival* and *survminer* packages for R V.4.0.3. Estimation of cumulative incidence functions from the competing risk scenario of time to AN progression and colectomy during follow-up was performed using R package *cmprsk*.

### *UC-CaRE* risk prediction model development using discovery data

The *UC-CaRE* web tool was created to make patient-specific AN risk prediction, using Shiny interface to R.^23^ The multivariate model above was embedded in a web tool that takes patient-specific features as user input, produces a cumulative AN risk curve into future years of follow-up and displays a Paling chart illustrating the patient’s individual risk (see online supplemental material for prognostic risk function derivation).

### Evaluation of *UC-CaRE* risk predictions in validation data

We evaluated the risk predictions produced by the *UC-CaRE* tool in the independent validation data set by computing the observed versus expected cumulative number of progressors to AN in the 13 years of follow-up data available after baseline index LGD. We assessed model discrimination using time-dependent area under the receiver operating characteristic curve (AUC), prediction error using Brier scores and calibration based on cumulative AN progression-specific hazards for overall and risk score-specific subgroups of the validation data (see online supplemental material for detailed methods on model performance metrics).

## Results

### Study patient clinical characteristics and outcomes

A total of 460 patients were followed for 2207 patient-years. There were 249 patients from St Mark’s Hospital (discovery cohort) and 211 patients in the multicentre validation cohort (figure 1) and detailed clinical characteristics were collected on each patient (online supplemental table S1, figure S1). In the discovery cohort, 7% (n=18/247) of the index LGD was invisible. Ninety percent (n=209/231) of the visible LGD was successfully resected endoscopically. After LGD diagnosis, patients had a median of four follow-up colonoscopies (IQR 2.0–7.0) and a median follow-up period of 5.1 years (IQR 2.3–8.5). Twenty percent (n=50/249) eventually had a colectomy performed due to neoplasia or symptomatic disease after the index LGD diagnosis. Over the follow-up period, 12.0% (n=30/249) progressed to either HGD or CRC and 7.2% (n=18/249) progressed to CRC.

There was significant heterogeneity in the clinicopathological characteristics between the discovery and validation cohorts as detailed in table 1. Despite this, there were no significant differences in the incidence rates of AN and CRC between the two cohorts. The incidence rates of AN per 100 patient-years for the discovery cohort (n=249), validation cohort (n=211) and the total cohort (n=460) were 2.2 (95% CI 1.5 to 3.0), 3.1 (95% CI 2.0 to 4.5) and 2.5 (95% CI 1.9 to 3.2), respectively. The total cohort incidence rate of AN per 100 patient-years was: 1.1 (95% CI 0.7 to 1.8) after successful endoscopic resection of unifocal visible LGD; 5.2 (95% CI 1.9 to 11.5) if unifocal visible LGD was not completely endoscopically resected; 7.0 (95% CI 3.7 to 12.1) if there was unifocal invisible LGD; 2.6 (95% CI 1.3 to 4.8) after successful endoscopic resection of multifocal LGD; and 19.3 (95% CI 10.5 to 32.8) if multifocal LGD was not endoscopically resected. Incidence rates of CRC followed similar trends (see online supplemental table S2).

**Table 1:** Clinical characteristics of patients with LGD for the discovery and validation cohorts

Clinical characteristics of included patients with LGD	Discovery cohort	Validation cohort	Difference between groups
	(n=249)	(n=211)	(P value)
Gender	(n=249)	(n=211)	0.276
Female	85 (34.1%)	62 (29.4%)	
Male	164 (65.9%)	149 (70.6%)	
Median age at index LGD diagnosis (years)	62.0 (IQR 53.5–69.0)	58.0 (IQR 48.0–68.0)	0.021
Median duration of UC at index LGD diagnosis (years)	22.0 (IQR 12.0–33.0)	16.0 (IQR 6.0–27.0)	**<0.001**
Colitis extent proximal to splenic flexure	225/249 (90.4%)	166/210 (79.0%)	**0.001**
Presence of concomitant PSC	13/248 (5.2%)	38/191 (19.9%)	**<0.001**
Exposure to 5-aminosalicylates	(n=238)	(n=74)	0.382
None documented	28 (11.8%)	9 (12.2%)	
0–10 years	53 (22.3%)	11 (14.9%)	
>10 years	157 (66.0%)	54 (73.0%)	
Exposure to immunomodulators	(n=237)	(n=75)	0.354
None documented	171 (72.2%)	48 (64.0%)	
0–10 years	43 (18.1%)	19 (25.3%)	
>10 years	23 (9.7%)	8 (10.7%)	
Exposure to biological therapy	9/236 (3.8%)	1/76 (1.3%)	0.282
Morphology of index LGD	(n=247)	(n=205)	**<0.001**
Polypoid	142 (57.5%)	133 (64.9%)	
Non-polypoid	87 (35.2%)	42 (20.5%)	
Invisible	18 (7.3%)	30 (14.6%)	
Visible index LGD size 10 mm or more	79/248 (31.9%)	68/202 (33.7%)	0.684
Location of index LGD	(n=243)	(n=209)	**0.001**
Distal to splenic flexure	115 (47.3%)	133 (63.6%)	
Proximal to splenic flexure	128 (52.7%)	76 (36.4%)	
Successful endoscopic resection of index LGD (judged by endoscopic criteria)	209/249 (83.9%)	139/207 (67.1%)	**<0.001**
Multifocal LGD at index diagnosis	61/249 (24.5%)	37/211 (17.5%)	0.069
Previous indefinite for dysplasia	12/249 (4.8%)	9/210 (4.3%)	0.785
Presence of a colonic stricture	8/249 (3.2%)	2/210 (1.0%)	0.098
Scarring/tubular/shortened colon	137/249 (55.0%)	29/208 (13.9%)	**<0.001**
Multiple postinflammatory polyps	88/241 (36.5%)	58/209 (27.8%)	0.048
Cumulative inflammation burden (CIB) score from 5 years preceding index LGD diagnosis	(n=177) 1.5 (IQR 0.0–3.0)		
Maximum severity of histological active inflammation in any colonic segment *at the same time or within previous 5 years* of index LGD diagnosis	(n=247)	(n=207)	0.006
Quiescent	105 (42.5%)	66 (31.9%)	
Mild	80 (32.4%)	59 (28.5%)	
Moderate	47 (19.0%)	55 (26.6%)	
Severe	15 (6.1%)	27 (13.0%)	
Maximum severity of histological active inflammation in any colonic segment *within 5 years after* index LGD diagnosis	(n=227)	(n=207)	0.003
Quiescent	117 (51.5%)	75 (36.2%)	
Mild	52 (22.9%)	54 (26.1%)	
Moderate	47 (20.7%)	53 (25.6%)	
Severe	11 (4.8%)	25 (12.1%)	
Chromoendoscopy use	202/245 (82.4%)	148/208 (71.2%)	0.004
Median number of colonoscopies performed in surveillance follow-up	4.0 (IQR 2.0–7.0)	2.0 (IQR 1.0–4.0)	**<0.001**
Median follow-up time after index LGD diagnosis (years)	5.1 (IQR 2.2–8.5)	3.1 (IQR 1.3–5.8)	**<0.001**
Metachronous LGD on follow-up	(n=249)	(n=209)	**<0.001**
None	84 (33.7%)	109 (52.2%)	
In same colonic segment	38 (15.3%)	31 (14.8%)	
In different segment	127 (51.0%)	69 (33.0%)	
Number of progressed to advanced neoplasia	30/249 (12.0%)	25/211 (11.8%)	0.948
Number of progressed to CRC	18/249 (7.2%)	15/211 (7.1%)	0.96
Colectomy surgery after LGD diagnosis	50/249 (20.1%)	38/211 (18.0%)	**0.001**

Statistical significance required p<0.002 with Bonferroni multiple testing correction (bold values).

CIB score was defined as sum of average scores between each pair of surveillance episodes multiplied by the surveillance interval in years.

CRC, colorectal cancer; LGD, low-grade dysplasia; PSC, primary sclerosing cholangitis.

A subanalysis of the index LGD cases that were considered unresectable or incompletely resected was performed to determine whether differences in AN progression between visible and invisible LGD were due to differences in the proportion proceeding to proctocolectomy. In the situation where the highest malignant potential lesion detected was unresectable or incompletely resected non-polypoid LGD, 55.8% (n=24/43) had proceeded to a proctocolectomy at time of censoring. In the situation where the highest malignant potential lesion detected was invisible LGD, proportionately fewer (39.6%; n=12/48) had proceeded to proctocolectomy at time of censoring (χ^2^(1, n=91)=9.1; p=0.003). Comparing the invisible LGD group and the unresected non-polypoid LGD group, there were no significant differences in the median time to proctocolectomy from index LGD diagnosis (11.5 vs 11.0 months, respectively) nor in the proportion of CRCs that were detected incidentally on colectomy (62.5% in both groups) rather than during surveillance.

### Predictors of progression of LGD to AN

Five variables were found to be significantly predictive of progression to AN on univariate analysis of the discovery set (table 2). Due to subjective inconsistencies in endoscopic reporting of macroscopic inflammation, two variables pertaining to histological inflammation status were used—presence of moderate or severe active histological inflammation (Nancy histological index^18^ grade 3 or 4 or equivalent) at the time of or within the previous 5 years of the index LGD diagnosis or the CIB score (the average histological inflammation score between each pair of surveillance episodes multiplied by the surveillance interval in years^19^). The former was first entered into a multivariate model with the three other variables (table 3; one patient was removed due to missing LGD size, two patients were removed for missing inflammation data). All four variables remained significant predictors of AN progression: size of any visible index LGD being 1 cm or greater (adjusted HR 2.7 (95% CI 1.2 to 5.9); p=0.014); unresectable or incomplete resection of the index LGD (adjusted HR 3.4 (95% CI 1.6 to 7.4); p=0.002); multifocal LGD at index diagnosis (adjusted HR 2.9 (95% CI 1.3 to 6.2); p=0.007); and presence of moderate or severe active histological inflammation at the time of or within the previous 5 years of the index LGD diagnosis (adjusted HR 3.1 (95% CI 1.5 to 6.7); p=0.003). Partially due to less patient data available on CIB (n=177), we found that a four-variable multivariate model using CIB as the alternative inflammation score variable was slightly less significant overall (online supplemental table S3) and as presence of moderate to severe active histological inflammation was more practical to calculate than CIB, we used the first model for downstream analyses.

**Table 2:** Univariate analysis for progression to advanced neoplasia (AN) within the discovery set

Variable	n (n=249)	HR (95% CI)	P value
Female sex	85/249	1.1 (0.5 to 2.3)	0.79
Age at LGD diagnosis (years)	(n=249)		
Less than 40	16	1	
40–59	84	0.7 (0.2 to 3.4)	0.708
60 or more	149	0.7 (0.2 to 3.1)	0.656
Duration of UC at LGD diagnosis (years)	(n=247)		
0–10	44	1	
11–20	70	3.4 (0.7 to 15.1)	0.116
>20	133	2.5 (0.6 to 10.6)	0.229
Presence of concomitant PSC	13/248	2.6 (0.8 to 8.6)	0.116
Patient exposure to 5-aminosalicylate medication	(n=238)		
None documented	28	1	
0–10 years	53	1.4 (0.3 to 5.4)	0.664
>10 years	157	1.2 (0.4 to 4.2)	0.729
Patient exposure to immunomodulator medication	(n=237)		
None documented	171	1	
0–10 years	43	2.4 (1.1 to 5.4)	0.032
>10 years	23	0.8 (0.2 to 3.6)	0.809
Macroscopic morphology of index LGD	(n=247)		
Polypoid	142	1	
Non-polypoid	87	2.6 (1.2 to 5.7)	0.016
Invisible	18	4.1 (1.3 to 12.9)	0.016
Visible index LGD size 10 mm or more	79/248	3.8 (1.8 to 7.9)	**0.0004**
Location of index LGD	(n=243)		
Distal to splenic flexure	115	1	
Proximal to splenic flexure	128	0.7 (0.3 to 1.4)	0.27
Index LGD not endoscopically resected or incomplete resection	40/249	4.7 (2.2 to 10)	**<0.0001**
Multifocal LGD at index diagnosis	61/249	3.2 (1.6 to 6.5)	**0.002**
Previous diagnosis of indefinite for dysplasia	12/249	4.3 (1.5 to 12.5)	0.007
Presence of a colonic stricture	8/249	5.5 (1.7 to 18.6)	0.006
Scarring/tubular/shortened colon	137/249	1.6 (0.7 to 3.3)	0.251
Moderate to severe histological active inflammation severity at time of or within previous 5 years of LGD diagnosis	62/247	3.6 (1.7 to 7.6)	**0.0006**
Cumulative inflammation burden (CIB)* (HR per 2-unit increase in CIB)	(n=177)	3.8 (1.8 to 8.0)	**0.0004**
Multiple postinflammatory polyps	88/241	1.5 (0.7 to 3.2)	0.26

Risk factors for LGD progression to high-grade dysplasia or colorectal cancer (univariate Cox regression analysis—30/249 AN). Statistical significance required p<0.003 with Bonferroni multiple testing correction (bold values).

*HR per 2-unit increase in cumulative inflammatory burden (equivalent to increase of 2 years of continuous mild, 1 year of continuous moderate or 8 months of continuous severe active disease).

LGD, low-grade dysplasia; PSC, primary sclerosing cholangitis.

**Table 3:** Multivariate model for progression to advanced neoplasia (AN) within the discovery set

Risk factor in final model	HR (95% CI)	P value
Visible index LGD size 10 mm or more	2.7 (1.2 to 5.9)	0.01400
Index LGD not endoscopically resected or incomplete resection	3.4 (1.6 to 7.4)	0.00190
Multifocal LGD at time of index LGD diagnosis	2.9 (1.3 to 6.2)	0.00749
Moderate or severe active histological inflammation in any colonic segment at time of or within previous 5 years of index LGD diagnosis	3.1 (1.5 to 6.7)	0.00305

Risk factors for LGD progression to high-grade dysplasia or colorectal cancer (multivariate Cox regression analysis). n=246 total patients included with complete data available, 29 progressed to AN. Score (log rank) overall p=9e-10 for model.

LGD, low-grade dysplasia.

We performed an additional analysis for model selection by including variables beyond the four described above that were found to have p<0.1 on univariate analysis. This included three additional variables for LGD—macroscopic morphology, previous diagnosis of indefinite for dysplasia and stricture—that were previously found to be associated with time to AN progression in patients with UC with LGD.^21^ Using an automated backward elimination selection algorithm based on the Akaike information criterion within R package *pec*, the same multivariate model was selected as was determined using our initial method described above, based on Bonferroni adjusted p values for inclusion of variables from univariate analysis. Multicollinearity was not detected for the four variables included in our final multivariate model (see online supplemental table S6).

The British Society of Gastroenterology guidelines outlining dysplasia management were first published in 2010.^20^ Since then, there has also been widespread adoption of chromoendoscopy, high-definition imaging and advanced polypectomy techniques. We first performed an internal validation of the full multivariate model by considering the individual risks predicted for the modern data alone (LGD diagnosed in 2010 and later; 115 patients with complete data on four risk factors), and computed the expected (E) number of cases using the cumulative hazard function from the Cox regression model. We found the model is well calibrated and accurately predicted the total number of 11 observed (O) future AN cases, with a standardised incidence ratio, O/E=0.99 (95% CI 0.50 to 1.78). To account for these more recent changes in practice, we also performed a stratified multivariate analysis for index LGD diagnosed pre-2010 and in year 2010 or later. Fitted models assigned very similar risks to all four predictor variables in both eras (online supplemental table S4) and estimated similar baseline cumulative hazards estimated for each era (online supplemental figure S2). Thus, the adoption of modern endoscopic techniques does not appear to have altered features that define LGD progression risk, possibly because the majority of LGD detected in the modern era remains low-risk lesions.

To externally validate the multivariate model’s predictions, we turned to the independent validation set. Estimated baseline hazard was similar in both cohorts (online supplemental figure S5). Individual patient risk scores for the validation set (23/198 AN progressors among patients with data on four predictors) were calculated and the predicted AN risk curves are illustrated in online supplemental figure S8. Risk prediction was similarly accurate in training and validation sets, and we found high discriminatory ability (AUC=0.89, online supplemental figure S6) and minimal prediction error (Brier score=0.068, online supplemental figure S7, table S7) by year 3 when applied to the independent validation set. For calibration, we computed the standardised incidence ratio, O/E, by comparing the number of observed (O) cases in the validation set versus the number of expected (E) cases as predicted by our model considering all follow-up (online supplemental table S8). We found O/E=1.01 (95% CI 0.64 to 1.52) confirming the model’s efficacy and predictive power, which was more accurate than predictions obtained using a Poisson regression approach (online supplemental table S9).

We also investigated the association of follow-up variables after baseline, such as occurrence of metachronous LGD or histological active inflammation score, with AN progression. A greater proportion of patients from the discovery cohort who developed metachronous LGD after the index LGD diagnosis progressed to AN (17.0%; n=28/165), compared with the patients who did not develop metachronous LGD (2.4%; n=2/84) (χ^2^(1, n=249)=11.2; p=0.001). Metachronous LGD was more likely to occur in those patients who already had presented with multifocal LGD at the time of the index LGD diagnosis (78.7%; n=48/61) rather than unifocal LGD (62.2%; n=117/188) (χ^2^(1, n=249)=5.6; p=0.018). Of the 28 patients with metachronous LGD who then went on to develop AN, most of the metachronous LGD had occurred in a different colonic segment to the index LGD (71.4%; n=20/28) rather than in the same segment (28.6%; n=8/28). Risk stratification determined at baseline by our model in the subset of patients who developed metachronous LGD remained significant (log rank p<0.0001, see online supplemental figure S10). Presence of histologically detected moderate or severe active inflammation within the 5 years following the index LGD diagnosis was not significantly associated with progression to AN (13.8%; n=8/58) compared with those patients who had quiescent or mild inflammation (8.9%; n=15/168) (χ^2^(1, n=226)=1.1; p=0.291). Over the follow-up period, most patients’ inflammation scores did not change (71.9%; n=161/224), but an equal proportion of patients demonstrated a decrease in their inflammation score from moderate-severe to quiescent-mild (13.8%; n=31/224) or an increase in their inflammation score from quiescent-mild to moderate-severe (14.3%; n=32/224). AN progression was not more evident in the patient group whose inflammation score increased to moderate-severe (3.1%; n=1/32) compared with the groups whose inflammation scores remained unchanged (9.3%; n=15/161) or decreased (19.4%; n=6/31) over the follow-up period (χ^2^(2, n=224)=4.8; p=0.089) (see online supplemental figure S11).

### Risk stratification with simple risk score in discovery and validation sets

We assigned a risk score to each patient based on the number of risk factors present (0–4 possible in total), combining patients with three or four risk factors due to low numbers. KM curves for the risk tiers (figure 2, online supplemental figure S3) in discovery versus validation sets confirmed very similar risk profiles in both cohorts (log rank p<0.0001 in both cohorts), which remained true when considering combined post-2010 data alone (online supplemental figure S4). Similar results were found when stratification was performed for five risk tiers (0–4) or three risk tiers (0, 1–2, 3+) (online supplemental figure S9).

We computed predictive values for all risk groups in the discovery set and then found similar results for predictive power in the validation set (online supplemental table S5). Reassuringly, the group with lowest risk score=0 (n=54) in the validation set had an NPV of 1 through all years of follow-up, that is, no patient in this group progressed to AN, thus we determined the lowest risk with perfect specificity using our model in this validation group. For the highest risk group, risk score=3+ (n=26), in the validation set we found PPV=12% by 6 months of follow-up, PPV=16% by year 1, PPV=35% by year 3 and PPV=48% by year 5.

Finally, we conducted a competing risk analysis for time to colectomy versus risk of developing AN. The HRs, based on the four risk factors above, were similar for both events (online supplemental figure S12). Thus, our findings suggest that our risk score predicts colectomy risk equivalent to predicting AN risk (online supplemental table S10 and online supplemental material).

### *UC-CaRE* risk prediction web tool development

**Figure 3:**
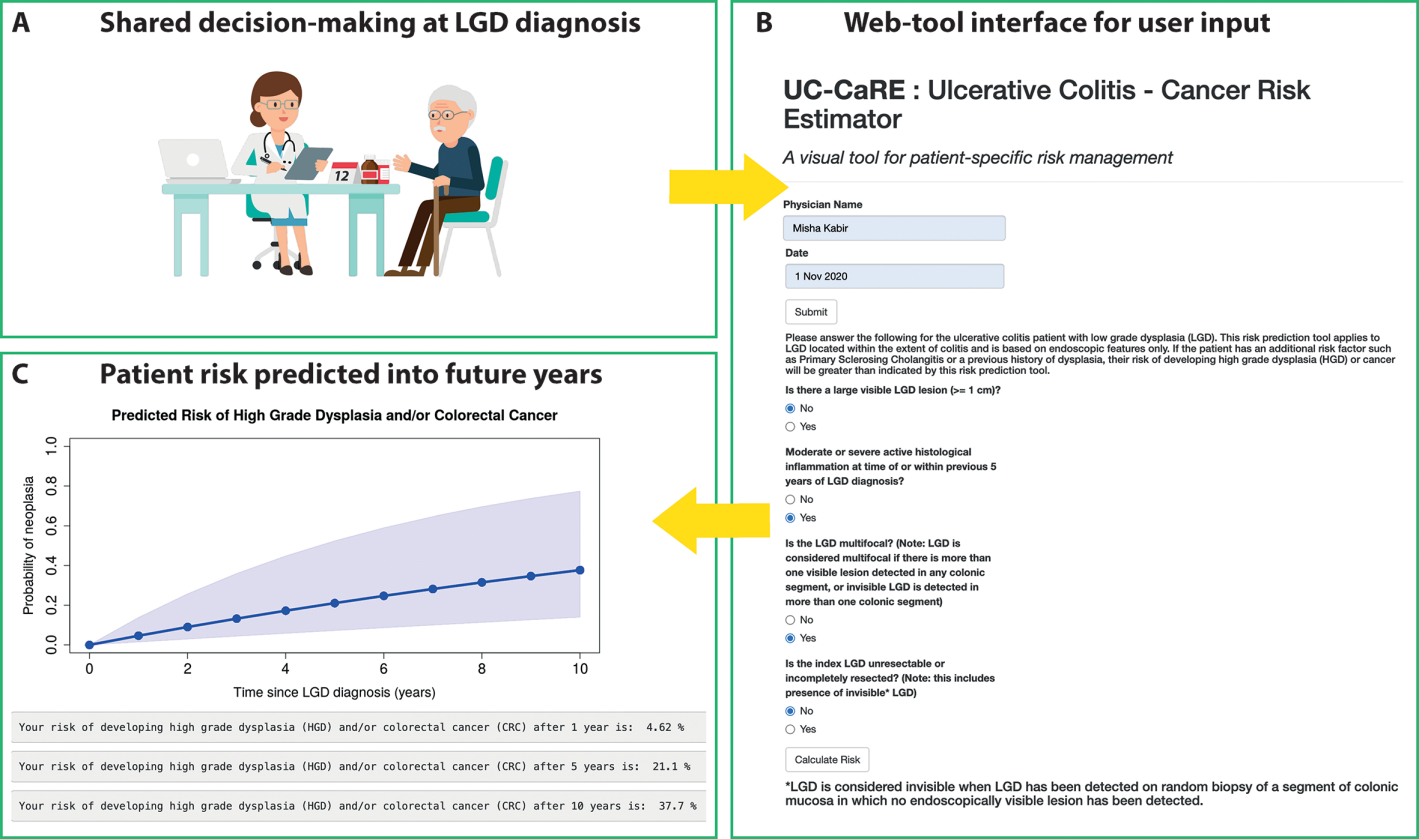
Ulcerative Colitis-Cancer Risk Estimator (*UC-CaRE*) clinical decision support web tool user pipeline. (A) Clinician records clinicopathological variables for patient at low-grade dysplasia (LGD) diagnosis (postresection, if performed) for shared decision-making consultation. (B) Simple interface in web tool to input patient characteristics. (C) Plot is created for predicted patient risk of progression to advanced neoplasia, as determined by the multivariate model, at each year of future follow-up up to 10 years, with percentages also provided for consideration.

**Figure 4:**
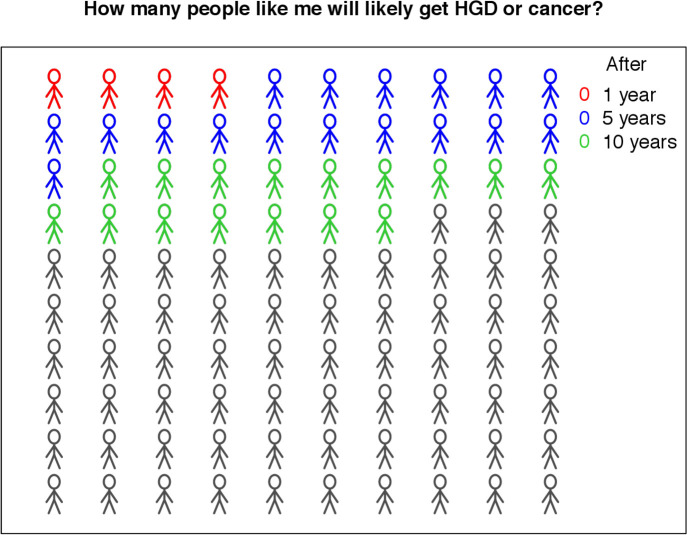
Ulcerative Colitis-Cancer Risk Estimator (*UC-CaRE*) online risk report. Paling charts provide a user-friendly display of the predicted cumulative risk of advanced neoplasia at 1, 5 and 10 years since low-grade dysplasia diagnosis/resection given patient characteristics. HGD, high-grade dysplasia.

We built a web tool named *UC-CaRE* to be used by a clinician to predict and display risk of AN for a patient with UC with LGD. The tool takes the four patient-specific variables included in the final multivariate model as user input, and computes the function *Risk(t)* for probability of AN progression at time *t* based on those variables and the baseline hazard (see the Materials and methods section and online supplemental material). Risk estimates are displayed as risk prediction curves (figure 3), and also demonstrated with the aid of a diagram of 100 patients with the same risk, coloured according to how many of the total will likely develop an advanced neoplasm in 1, 5 and 10 years (figure 4). This latter type of visual aid (also known as a Paling chart) can be helpful for patients to understand the meaning of a probability of cancer occurrence by viewing a simple diagram of predicted outcomes for 100 similarly at-risk patients with UC (figure 4). The ‘risk report’ summarising the *UC-CaRE* output can be downloaded as a PDF file for ease of display and recording purposes.

## Discussion

We designed and validated a cancer risk prediction tool *UC-CaRE* using multicentre data from patients with UC diagnosed with LGD. We intend this tool to be used by clinicians to communicate personalised AN risk and to be used by patients to make a more informed choice to either accept colectomy or continue endoscopic surveillance.

Cancer risk communication in the context of LGD is particularly challenging as there remains much uncertainty about individualised risk. Providing evidence-based and individualised numerical CRC risk estimates has been reported by patients to facilitate shared decision-making^13 15^ and prevents avoidance of decision-making.^14^ Personalised pictorial depiction of risk particularly aids patient comprehension and engagement.^14 24^ *UC-CaRE* provides a convenient way for a clinician to demonstrate a patient’s predicted absolute risk of AN using line graphs and Paling chart automatically created by the web tool. For example, *UC-CaRE* predicts the 5 and 10-year risks of a patient with UC with a 15 mm non-polypoid endoscopically unresectable LGD lesion on a background of moderate active inflammation (ie, has three risk factors) to be 53% and 78%, respectively, and the high risk would appear visually apparent on the associated Paling chart. In future iterations with larger data sets including longitudinal information on when a new risk factor has appeared, that is, multifocal LGD has developed, we hope to further develop and test the *UC-CaRE* tool to recalculate future CRC risk predictions. Importantly, the tool is able to predict with 100% specificity the patients at the lowest risk of progression to cancer and for whom continued surveillance rather than prophylactic colectomy can be confidently recommended. Finally, model prediction remained highly accurate in the validation set, even when only considering LGD diagnosed in the most recent post-2010 time period.

We recognised that censoring patients at colectomy, before they have had time to progress to AN, was a competing risk for patients in our study. Our results confirm that the risk of both events (colectomy or AN progression) was similar, even when stratified by AN risk group. Thus, colectomy decisions made in the absence of an AN diagnosis are likely preventing AN development, with minimal overtreatment. Thus, it is reasonable to suggest that progression to AN could have been prevented by earlier colectomy in patients identified as high risk by the *UC-CaRE* tool.

A number of clinicopathological variables have been reported by previous studies to be associated with AN progression after LGD diagnosis: patient-specific characteristics such as age ≥55 years, male sex, follow-up at an academic (vs non-academic) medical centre, concomitant PSC and endoscopic characteristics of the index LGD such as non-polypoid morphology, invisibility (ie, detected on random mucosal biopsy with no associated visible lesions), size greater than 10 mm, multifocality, presence of a stricture and distal location.^21 25 26^ The St Mark’s patient data set used in the discovery set overlaps with a previously reported cohort study of 172 patients with UC with LGD diagnosed between 1993 and 2012 by Choi *et al*.^21^ In this older study, lesion size greater than 10 mm (HR 10.0; 95% CI 4.3 to 23.4), multifocality (HR 5.0; 95% CI 1.9 to 7.8) and non-polypoid morphology (HR 16.5; 95% CI 6.8 to 39.8) were significant predictive factors for AN progression. In Fumery *et al*’s meta-analysis^26^ of LGD outcomes, multifocality (OR 3.5; 95% CI 1.5 to 8.5) and invisibility (OR 1.87; 95% CI 1.04 to 3.36) were significant predictive factors for AN development. Strengths of our more recent study include the fact that it is the largest cohort study to date to have additionally evaluated the impact of endoscopic resectability on LGD prognosis. We note that endoscopic unresectability has not been assessed in previous studies,^21 25 26^ which is an important omission given that it is an indication for colectomy surgery to prevent CRC progression.^4–7^ Another strength of the present study is the inclusion of LGD cases only diagnosed within the 21st century to better reflect modern surveillance practices. Chromoendoscopy was adopted into routine surveillance practice at all the study centres from the beginning of the study period and true high-definition imaging processors have been available from 2012 onwards. We have demonstrated indistinguishable progression risk curves when comparing long-term AN incidence between truly invisible LGD and endoscopically unresectable or incompletely resected visible LGD in the validation data (online supplemental figure S13). These findings suggest that caution should be exercised when delaying proctocolectomy for invisible dysplasia.

We have reported incidence rates of LGD progression to AN and CRC of the total cohort (n=460) as being 2.5 and 1.5 per 100 patient-years’ follow-up, respectively. The CRC rate is similar to that found in a Dutch population-based cohort study of 4284 patients with IBD-LGD (1.4 per 100 patient-years).^25^ A meta-analysis^26^ reported lower AN rates but there was substantial calculated between-study heterogeneity and studies that included LGD proximal to the colitis extent. There is a paucity of cohort studies reporting on long-term CRC incidence rates after endoscopic resection of non-polypoid dysplasia. Our incidence rate of CRC progression after endoscopic resection of both unifocal polypoid and non-polypoid dysplasia (0.6 per 100 patient-years) is very similar to the pooled incidence calculated in a meta-analysis of endoscopically resected polypoid-only dysplasia (0.5 per 100 patient-years).^27^

It is important to note the limitations of our study. This was a retrospective study relying on the accuracy of the available medical documentation, and incomplete medical records meant that other important risk factors for CRC development, such as smoking history and family history of CRC, could not be included in the risk prediction model. Overall incidence of LGD and LGD progression to AN in the IBD population is low,^21^ resulting in a model that has been developed on a relatively modest number of patients with AN events (n=30) in the discovery cohort. The modest number of patients with PSC (n=13) available for use in the discovery cohort may explain why this variable was not significantly associated with AN progression. It is well recognised that PSC is an important risk for AN progression in UC (OR 3.4; 95% CI 1.5 to 7.8),^26^ therefore it is likely *UC-CaRE* will underestimate AN risk in patients with PSC and should not be used for this subset of patients. These limitations in population size and event rate can only be overcome by conducting larger prospective studies. However, we note that increased AN risk in PSC was still predicted appropriately for the 38 patients with PSC in the validation set because they had elevated risk predicted based on the same risk factors as the total UC population (eg, patients with PSC had double the proportion of patients in the highest estimated risk group than that of the total UC population). By only including tertiary IBD centres in both discovery and validation cohorts, our results may also be limited by selection bias. However, we demonstrated significant heterogeneity between the two cohorts reflective of the variation in patient demographics and clinical practice likely to be seen outside of tertiary centres. Despite this heterogeneity we still found that the *UC-CaRE* model can accurately predict risk groups. We have also discussed above that the CRC incidence rate found with our study cohort was similar to that found in a population-based cohort study which included non-academic centres.^25^ We therefore believe that the *UC-CaRE* model can be extrapolated to include patients from non-tertiary centres. A further unavoidable limitation of this study is that surveillance colonoscopy in itself is not a perfect diagnostic test in excluding all AN. Therefore, it is conceivable that some of the patients, who were not categorised as proceeding to an AN event or colectomy and were censored at their last colonoscopy, may have had in fact an undetected focus of AN at that colonoscopy. However, the high proportion of patients in the discovery cohort who had chromoendoscopy (82.4%) with a median of four follow-up colonoscopies performed per patient reassures us that missed AN at time of censoring was less likely.

The significant proportions of the total cohort with at least one episode of moderate to severe histological active inflammation and the low exposure to anti-tumour necrosis factor (TNF)-alpha agents (the only biological drug exposure in this cohort) highlight how clinical practice and therapeutic targets have evolved over the time period of this study. The National Institute for Health and Care Excellence approved the use of anti-TNF-alpha agents in the UK for chronic moderate to severe UC in 2015. Vedolizumab, ustekinumab and tofacitinib have since been approved for use in UC but none of our study patient cohorts were exposed to these. The benefits of achieving strict histological remission rather than clinical remission have also been realised in recent years with a shift in treatment targets.^28^ We noted a trend for exposure to 5-aminosalicylates (less than 10 years’ duration) or immunomodulators to be associated with an increased risk of AN progression on univariate analyses. However, we believe that medication exposure in our study was a proxy for underlying chronic inflammation rather than AN progression occurring as a result of the medication exposure itself. Recent studies have suggested that immunomodulator^29^ and anti-TNF exposure^30^ may in fact be associated with lower cancer risk, but it is uncertain as to whether this is indirectly a result of achieving inflammatory control and mucosal healing or a direct chemoprotective effect. Further prospective data are required to understand the long-term impact of medical therapy, particularly biological drugs, on CRC risk in IBD.

## Conclusion

Our large multicohort retrospective validation study confirms that patients with large, unresected and multifocal LGD and recent moderate/severe active inflammation are at the highest risk of developing AN. We have derived and validated a simple-to-use web tool, *UC-CaRE* (www.UC-CaRE.uk), for the calculation of personalised patient-specific HGD and/or CRC risk in individuals with UC and LGD. We believe the tool will be useful as an adjunct used by clinicians when managing CRC risk together with their patients. Further validation of the *UC-CaRE* tool using prospective data from non-tertiary centres will help test its applicability for generalised use and to reflect evolving clinical practice.

## Data Availability

Data are available upon reasonable request. All essential outputs from model construction are included in the main text (table 3) and supplemental materials (baseline hazard and variance-covariance matrix for multivariate model provided, online supplemental table S11) to enable implementation of our analysis using other data. Sharing of individual-level data requires successful completion of a data sharing agreement with St Mark’s Hospital, in accordance with our ethical approval.
